# Supplementary material for: Content-rich biological network constructed by mining PubMed abstracts
Source: BMC Bioinformatics. 2004 Oct 8;5:147. doi: 10.1186/1471-2105-5-147 (PMC528731; doi:10.1186/1471-2105-5-147)
Supplement: Additional File 5 — The original Chilibot query results of the term "long-term potentiation (LTP)" and 22 other terms, limiting the latest references analyzed to the years 1990, 1995, 2000, and 2004. [file 1471-2105-5-147-S5.bz2 › chilibotAdditionalFile5/ltp2004/html/PLC_CAMKII.html]

 


 **PLC** and **CAMKII** 
  
Found 5 abstracts in PubMed,  **5 abstracts were retrieved and analyzed**.  


---

 Search Google  |
 PDF files only 
|  EDU domain only 

---

**Interactive relationship** (e.g. stimulation, inhibition, etc)

- It is thus concluded that InsP3 3 kinase up regulation occurs through activation of  **PLC**  coupled serotoninergic receptors and requires the phosphorylation of the enzyme by the ubiquitous multimeric protein kinase  **CaMKII** .  Ref: 9835216 Neurosci Lett, 1998

- :-)

  **Parallel relationship** (e.g. studied together, co-existance, homology, etc.)

  - The response to S100A12 was abolished by inhibitors of phospholipase C  **PLC** , protein kinase C PKC, calcium flux, calcium calmodulin dependent kinase II  **CaMKII**  or mitogen activated protein kinase kinase MEK.  Ref: 11723169 J Neurochem, 2001
  - Also, the release of CRF depends on the  **PLC**  beta to InsP3 R axis and  **CaMKII**  activity but also entails the participation of a calcium independent PKCs.  Ref: 10919265 Endocrinology, 2000
  - In short, this study in astrocytes demonstrates that under physiological calcium and adenylate cyclase stimulation an elevation of cAMP production is achieved that is mediated by  **PLC**  IP 3 and  **CaMKII**  dependent pathways and results in the release of endogenous adenosine which acts at G s protein coupled A 2A receptors.  Ref: 12213273 Neuropharmacology, 2002
  - Pre incubation with the phospholipase C  **PLC**  inhibitor U73122 10 microM, L type calcium channel blockers nifedipine 1 microM and nimodipine 1 microM, the calmodulin kinase II  **CaMKII**  inhibitor KN 62 10 microM or pertussis toxin 100 ng ml inhibited this potentiation.  Ref: 12213273 Neuropharmacology, 2002
  - The stimulation of CRF synthesis by NPY seems to depend mainly on a  **PLC**  beta to InsP3 R axis and on  **CaMKII**  activity.  Ref: 10919265 Endocrinology, 2000
  - These results demonstrate that Ang II produces an inhibition of I Kv in CATH.a cells, via an intracellular pathway that includes  **PLC** , PKC, and  **CaMKII** .  Ref: 14550259 Biochem Biophys Res Commun, 2003
